# Disease-Relevant Preoperative Serum miRNA Levels in Papillary Thyroid Cancer

**DOI:** 10.3390/biology15080626

**Published:** 2026-04-16

**Authors:** Olga Bourogianni, Eliza Tsitoura, Konstantinos Sifakis, Nikolaos Kapsoritakis, Alexander Karatzanis, Maria Doulaptsi, Katerina Antoniou, Sophia Koukouraki, Emmanuel Prokopakis

**Affiliations:** 1 Department of Nuclear Medicine, University of Crete School of Medicine, 71500 Heraklion, Crete, Greece; 2 Laboratory of Molecular and Cellular Pneumonology, Department of Respiratory Medicine, University of Crete School of Medicine, 71003 Heraklion, Crete, Greece; e.tsitoura@med.uoc.gr (E.T.);; 3 Department of Otorhinolaryngology-Head and Neck Surgery, University of Crete School of Medicine, 71500 Heraklion, Crete, Greece; 4 Laboratory of Translational Otorhinolaryngology Research, University of Crete School of Medicine, 71500 Heraklion, Crete, Greece

**Keywords:** papillary thyroid cancer, serum miRNAs, diagnosis, prognosis, radioiodine treatment

## Abstract

Background: Papillary thyroid cancer (PTC) is the most common form of thyroid malignancy, with an incidence that has been steadily rising globally. Early and accurate diagnosis remains crucial for effective treatment and improved outcomes. MicroRNAs (miRNAs), small non-coding RNA molecules that regulate gene expression, have emerged as promising biomarkers in cancer research due to their stability and accessibility in serum. In this pilot study we compared the expression of 84 consistently reported, malignancy-associated serum miRNAs in patients with PTC (PTC group) and benign thyroid disease (Control group) as potential PTC markers. Methods: A focused panel containing primer assays for 84 human miRNAs that are consistently reported in the literature as being detectable and differentially expressed in serum in various organ-specific cancers was used to measure miRNA levels in serum samples from PTC (*n* = 8) and benign thyroid disease (*n* = 6) patients prior to thyroidectomy. Results: Among the 84 miRNAs analyzed, a panel of ten miRNAs showed numerical trends of differential expression between the two groups, including three upregulated (hsa-miR-150-5p, hsa-miR-21-5p, hsa-miR-23a-3p) and seven downregulated miRNAs (hsa-miR-17-5p, hsa-miR-17-3p, hsa-miR-200c-3p, hsa-miR-296-5p, hsa-miR-574-3p, hsa-miR-885-5p, hsa-miR-130-3p). The serum expression levels of hsa-miR-23a-3p were markedly elevated in patients with malignant nodules compared with those with benign lesions, while hsa-miR-574-3p was significantly downregulated in the PTC group. Conclusions: These findings warrant further investigation of hsa-miR-23a-3p and hsa-miR-574-3p in larger cohorts of patients with PTC to validate their potential clinical relevance.

## Simple Summary

Our research focused on finding new, easy-to-measure biological markers for papillary thyroid cancer (PTC). In this pilot study, we examined microRNAs (miRNAs)—small RNA molecules that control gene expression—in the blood of patients. MiRNAs are potential markers because they are stable and easily accessible through a simple blood draw. We analyzed the levels of 84 disease-specific circulating miRNAs in the serum of two groups: patients with confirmed PTC and patients with benign thyroid disease (Control group). All samples were taken before surgery. We identified 10 miRNAs whose levels differed noticeably between the cancer patients and the control patients. Among these, two specific miRNAs, hsa-miR-574-3p and hsa-miR-23a-3p, showed promise as potential serum biomarkers for PTC diagnosis.

## 1. Introduction

Papillary thyroid cancer (PTC) is the most common histological type of differentiated thyroid cancer, making up 75–85% of the total incidence of thyroid cancer. In the last three decades, the incidence of PTC has increased steadily worldwide [1]. However, between 2013 and 2022, the incidence of newly diagnosed cases of thyroid cancer per 100,000 population decreased by an average of 0.8% per year [2]. Despite the high five-year overall survival rate, relapse can occur. Several genetic loci are associated with the prognosis and occurrence of PTC, including tyrosine kinase receptor genes rearranged and point mutations in the RAS and BRAF proteins [3,4]. The *BRAF*V600E mutation is considered the most common and early genetic event, occurring in 29–87%. However, it is not sufficient as an independent predictive factor and should be combined with other molecular and clinico-pathological risk factors [5].

In the last few years, a significant interest in the discovery of novel diagnostic, prognostic and predictive biomarkers in cancer, especially microRNAs (miRNAs), has been noted [6]. It has been noted that miRNAs comprise only 3% of the human genome but regulate more than half of the protein-coding genes, which are involved in the regulation of processes like development, organogenesis, cell cycle, and metabolism [7].

The first study on the involvement of miRNAs in thyroid tumorigenesis was published in 2005, when it was found that a group of five miRNAs, including hsa-miR-221, hsa-miR-222 and hsa-miR-146 among the top upregulated, were able to distinguish unequivocally PTC from normal thyroid tissue. Additionally, hsa-miR-221 was found to be upregulated in unaffected thyroid tissue in several PTC patients, suggesting the early involvement of this miRNA in the process of thyroid carcinogenesis [8].

The significant role of these molecules in the pathogenesis of PTC has been confirmed in the following studies [9,10,11,12,13,14,15,16]. Graham et al. identified four miRNAs, including hsa-miR-146a-5p, hsa-miR-199b-3p, let7b-5p and hsa-miR-10a-5p, which showed statistically significant differences when the serum of patients with PTC was compared with those with benign nodules [17]. Lee et al. showed that hsa-miR-146b and hsa-miR-155 expression levels helped in discriminating benign from malignant thyroid lesions [18]. In another study, Zhang et al. analyzed the expression levels of three reportedly upregulated miRNAs (hsa-miR-222, hsa-miR-221 and hsa-miR-146b) [11]. More recently, Armos et al. performed comprehensive miRNA profiling in tissue specimens from PTC patients, identifying a distinct set of dysregulated miRNAs associated with tumor pathogenesis and clinicopathological features, thus highlighting the potential of tissue-based miRNA signatures as diagnostic and prognostic biomarkers [19].

We performed an exploratory analysis of 84 serum miRNAs in preoperative samples from patients with PTC and benign thyroid nodules. This analysis identified hsa-miR-23a-3p and hsa-miR-574-3p as consistently detectable, pathophysiologically relevant serum miRNAs. Our findings support the need for further validation of these candidate miRNAs in larger PTC patient cohorts to determine their clinical utility as biomarkers.

## 2. Materials and Methods

### 2.1. Patients

This prospective study included 24 consecutive patients who underwent total thyroidectomy at the Department of Otorhinolaryngology–Head and Neck Surgery at the University Hospital of Crete between August and December 2017.

**Figure 1:**
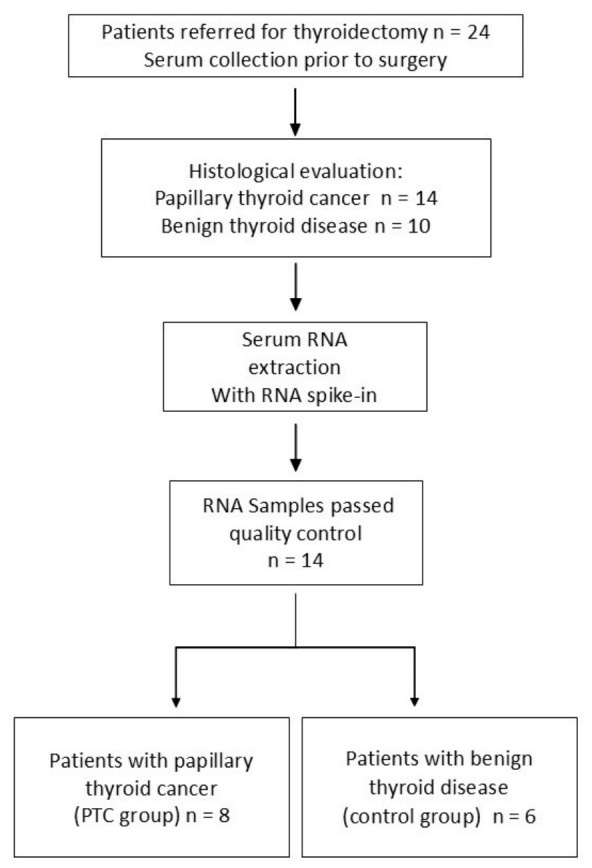
Flow chart depicting patient selection for the study cohort.

Patients diagnosed with PTC (*n* = 14) or benign thyroid nodules (*n* = 10) were included in the study (Figure 1). Patients with other malignancies or distant metastases were excluded. The study was carried out in accordance with the Declaration of Helsinki, and its protocol received approval from the Ethics Committee of the University Hospital of Heraklion (IRB No. 3691/29-03-2017, Decision 657).

Serum Thyroglobulin (Tg) assay, radioimmunometric assay of circulating anti-Tg antibodies, and high-resolution grayscale and color Doppler sonography of the thyroid bed and cervical lymph node compartments were all included in the post-treatment surveillance, which included follow-up visits every one to two years. At six months, twelve months, and five years post-radioiodine treatment (RAIT), a whole-body I-131 SPECT/CT scan (131I SPECT/CT WBS) was performed after the administration of 5 mCi I-131. The absence of thyroid tissue (no I-131 uptake) in the thyroid bed on the 131I SPECT/CT WBS and serum Tg levels less than 2 ng/mL under thyroid-stimulating hormone (TSH) stimulation were the criteria for successful ablation. Excellent clinical outcome was defined as the absence of biochemical or structural evidence of disease evaluated by ultrasonography, 131I SPECT/CT WBS, and serum Tg levels five years following total thyroidectomy and RAIT. All PTC patients were disease-free 5 years post-RAIT.

### 2.2. Sample Collection

Peripheral venous blood samples were collected into tubes without anticoagulant one day prior to surgery. Informed consent was obtained in written form. Coagulated samples were centrifuged at 2500 rpm for 10 min. Serum was carefully collected in 1.5 mL RNase-free tubes and immediately stored at −80 °C till further processing.

### 2.3. RNA Extraction and Quality Control

Frozen serum samples were thawed and centrifuged at 11,000× *g* for 3 min to remove any remaining cells or particles. Next, 200 µL of serum was added with 8.8 × 10^8^ copies of cel-miR-39 as a spike-in control. Subsequently, small RNA was isolated using the NucleoSpin miRNA Plasma Kit (Macherey-Nagel, Düren, Germany; Cat. No. 217184) according to the manufacturer’s instructions. RNA was eluted in 14 µL volumes and kept frozen at −80 °C until analysis. For reverse transcription, 2 µL of RNA samples were used according to the manufacturer’s protocol using the TaqMan miRNA Reverse Transcription Kit (Thermo Fisher Scientific, Waltham, MA, USA). The cDNA was diluted 1:3, and 2 µL of diluted cDNA was used in real-time quantitative PCR (RT-qPCR) in 10 μL reactions, using Taq-Man Universal Master Mix II, no UNG, performed on a Stratagene Mx3000P instrument (Agilent Technologies, Santa Clara, CA, USA). Thermal cycling parameters: Initial hold at 95 °C for 10 min, followed by 40 cycles of 95 °C for 15 s and 60 °C for 1 min. TaqMan Assay ID # 200 specific for cel-miR-39 was used according to the manufacturer’s instructions for assay performance. Samples with cel-miR-39 Ct values above 25 were removed from analysis, leaving 14 samples (6 benign, 8 PTC), as shown in Figure 1.

### 2.4. miRNA Quantification

A total of 1.5 μL RNA was used in reverse transcriptase and quantitative PCR reactions using the miScript II RT Kit (Qiagen, Hilden, Germany, Cat. No. 218160), miScript SYBR^®^ Green PCR Kit (Qiagen, Hilden, Germany, Cat. No. 218073) and Stratagene Mx3000P instrument (Agilent Technologies, Santa Clara, CA, USA).

Quantification of miRNAs was carried out using the Serum & Plasma miScript miRNA PCR Array panel (Qiagen, Hilden, Germany, MIHS-106ZA), a focused panel containing primer assays for 84 human miRNAs that are consistently reported in the literature as being detectable and differentially expressed in serum, plasma and other extracellular bodily fluids. These miRNAs are selected for their relevance to various organ-specific cancers (Supplementary Table S1).

Analysis of miRNA serum levels was performed using the GeneGlobe Data Analysis Center-Qiagen (https://geneglobe.qiagen.com/us/analyze (accessed on 10 April 2026)). Samples with a value of Ct > 36 were considered negative. Fold change was calculated with the 2^−ΔΔCt^ method as recommended by the GeneGlobe platform. In brief, average Cts of the 84 miRNAs in the PTC and Control groups were calculated. For each sample/miRNA assay, ∆CT was calculated by subtracting each assay’s CT value from the Global CT Mean of expressed assays per corresponding plate as a normalizer. Each assay-specific ∆CT value was averaged across the PTC and control samples for an AVG ∆CT. The assay-specific AVG ∆CT values in each test group were subtracted by the corresponding Control group values for a ∆∆CT. Finally, fold change was calculated as log_2_2^(−∆∆CT)^.

### 2.5. Statistical Analysis

Statistical analysis on differential expressions of serum miRNAs between the PTC and Control groups were performed by the GeneGlobe platform. *p*-values were calculated based on a Student’s *t*-test of the 2^(−AVGΔCt)^ values for each miRNA in the Control and PTC groups. miRNAs with >2-fold change and *p* < 0.05 were considered statistically significant, whereas *p*-values between 0.05 and 0.1 were considered to indicate nominal trends.

## 3. Results

### 3.1. Patient Demographics

Demographic data and clinicopathological characteristics of the patients are shown in Table 1. Six patients had no malignant disease following histological examination, and this group was referred to as the Control group. This group included patients with thyroid nodules: two (2) patients with a single-nodule-Bethesda III, three (3) patients with single-nodule-Bethesda IV according to preoperative ultrasound findings, and one (1) patient with multinodular goiter.

The PTC group included eight (8) patients with pathological findings of papillary thyroid carcinoma.

The mean age of PTC patients was higher than the patients in the Control group (Student’s *t*-test, *p* = 0.01), while the majority of patients in both groups were female as expected. The majority of PTC patients had multifocal nodules (6/8), and the diameter of the lesions was smaller than 10 mm in 87.5% of PTC patients. Additionally, according to the histopathological findings, lymph node metastasis was found in only 1/8 patients. No distant metastasis was found.

### 3.2. miRNA Expression Levels

We analyzed serum levels of 84 miRNAs (Supplementary Table S1) for differences in expression between PTC and benign thyroid lesions. Sixty-five miRNAs showed a numerical downregulation and 19 miRNAs were numerically upregulated (Supplementary Table S2).

**Figure 2:**
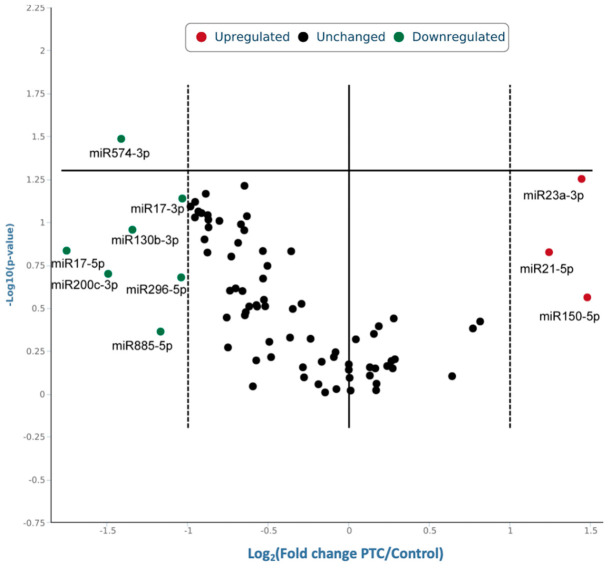
Volcano plot of the relative expression of 84 miRNAs in the serum of PTC patients relative to the benign group. Ten miRNAs were expressed in PTC group with more than a 2-fold difference compared with Control group. Green color represents seven miRNAs that were downregulated (hsa-miR-17-5p, hsa-miR-17-3p, hsa-miR-200c-3p, hsa-miR-296-5p, hsa-miR-574-3p, hsa-miR-885-5p, hsa-miR-130-3p) and red represents three miRNAs that were upregulated (hsa-miR-150-5p, hsa-miR-21-5p, hsa-miR-23a-3p).

Comparison of the average serum miRNA expression levels between the PTC group and the Control group showed that three miRNAs were expressed at more than 2-fold higher levels, whereas seven miRNAs were expressed at more than 2-fold lower levels in the PTC group compared to the controls. hsa-miR-150-5p, hsa-miR-21-5p, and hsa-miR-23a-3p are upregulated, and hsa-miR-17-5p, hsa-miR-17-3p, hsa-miR-200c-3p, hsa-miR-296-5p, hsa-miR-574-3p, hsa-miR-885-5p, and hsa-miR-130-3p are downregulated. (Table 2 and Figure 2). hsa-miR-574-3p was significantly downregulated in the PTC group (*p* = 0.032). The marginal significance zone was defined as *p*-values between 0.05 and 0.1, indicating nominal trends. hsa-miR-23a-3p exhibited a trend for upregulation (*p* = 0.055), while hsa-miR-17-3p tended to be higher in the Control group. The above-described trends in miRNA expression levels were also observed in the analysis of a smaller age-matched cohort of Control and PTC groups as shown in Supplementary Table S3.

## 4. Discussion

Motivated by the rising incidence of PTC in Crete [20,21], this pilot study investigated the serum expression profiles of a wide range of disease-relevant mature miRNAs in a small exploratory cohort of PTC patients versus those with benign thyroid disease. The identification of a circulating miRNA signature that can effectively discriminate PTC from benign disease is clinically significant, as its integration with ultrasound and Fine-Needle Aspiration (FNA) findings could potentially obviate the need for a thyroidectomy. The primary objective of this pilot study was to identify a panel of circulating molecular markers capable of achieving this discrimination.

Our findings represent preliminary evidence for the potential clinical utility of circulating miRNAs in PTC, highlighting candidates for further study in larger cohorts. In the 84 miRNAs assessed, the expression of 10 miRNAs was differentially expressed in the serum of PTC patients compared to the Control group, with more than a 2-fold difference. hsa-miR-150-5p, hsa-miR-21-5p, and hsa-miR-23a-3p were numerically upregulated, while hsa-miR-17-5p, hsa-miR-17-3p, hsa-miR-200c-3p, hsa-miR-296-5p, hsa-miR-574-3p, hsa-miR-885-5p and hsa-miR-130-3p were downregulated. In this exploratory study, we identified two serum miRNAs, hsa-miR-23a-3p and hsa-miR-574-3p, that showed potential to differentiate the two groups of patients, while hsa-miR-17-3p exhibited a nominal trend.

miRNAs show tissue specificity, and their changes can be traced not only in the tissues but also in the blood, as well as in other body fluids. They enter the extracellular environment in the form of exosomes or other microvesicles as well as in the form of protein complexes, transported from one cell or organ to another, thus helping in intercellular as well as inter-organ communication [22]. This method of transfer helps in the stability of circulating miRNA molecules in plasma and serum. It has also been seen that miRNAs are resistant to RNase activity, as well as extreme acidic and alkaline pH and temperature [10,11]. Circulating miRNAs have a great advantage of being stable and non-invasive as diagnostic biomarkers for PTC.

Fluctuations of circulating miRNA levels have been associated with thyroid dysfunction [23]. These markers can be analyzed in serum, potentially providing a fast, minimally invasive, safe and reproducible tool to discriminate malignant and benign thyroid disease [24]. Numerous studies have highlighted the pivotal role of miRNAs in the pathogenesis of PTC, as well as their potential as promising molecular biomarkers [23,24,25,26]. A large number of miRNAs seem to be involved at different stages of this biological cascade. In fact, more than 100 distinct PTC-related miRNAs have been reported in the literature to play either an oncogenic role, as oncogenic microRNAs (“oncomiRs”), or a tumor-suppressive role, as tumor suppressors.

Limited data exist on the role of hsa-miR-574-3p and hsa-miR-23a-3p in thyroid cancer; however, deregulation of these miRNAs is associated with other types such as gastric, bladder and ovarian cancer. hsa-miR-574-3p has been described as a tumor-suppressing miRNA in various cancers via targeting *ADAM28*. Su et al. noted that the reduced expression levels of hsa-miR-574-3p were mainly found in early stages, as well as in highly differentiated gastric cancer tissue, making this miRNA a promising tool for the detection of early stages of gastric cancer [27]. hsa-miR-574-3p expression was downregulated in bladder cancer cell lines, indicating that it could be a candidate tumor-suppressive miRNA in human bladder cancer [28]. Additionally, the tumor-suppressive function of hsa-miR-574-3p has been reported also in ovarian cancer, as underexpression in ovarian cancer tissue samples compared with adjacent tumor tissues [29].

In thyroid cancer, Borrelli et al. were the first to report the potential clinical role of hsa-miR-574-3p. They reported that hsa-miR-574-3p seems to be useful in differentiating noninvasive follicular thyroid neoplasms with papillary-like nuclear features (NIFTPs) and follicular adenomas. Especially, this miRNA in tissue was significantly downregulated in NIFTPs compared with follicular adenomas [30]. Similarly to our study, serum hsa-miR-574-3p was downregulated in lesions with nuclear features of PTC. To our knowledge, no study has mentioned the expression of serum hsa-miR-574 in PTC patients. Take into consideration the above and our study; the downregulation of serum hsa-miR-574-3p in PTC merits further investigation as a potential diagnostic biomarker.

Differential expression of hsa-miR-23a has been found across different types of cancer and participates in common aspects of carcinogenesis by targeting *FGF2* or *RUNX2* and dampening PI3K–AKT signaling [31]. Head and neck, respiratory, and digestive system carcinomas often demonstrate an over-expression of hsa-miR-23a. In contrast, hsa-miR-23a is downregulated in cancers of the genitourinary system and myelogenous leukemia. A large meta-analysis revealed that overexpression of hsa-miR-23a in tissue was associated with poor survival in various types of cancer, like diffuse B-cell lymphoma, hepatocellular carcinoma, gastric cancer, laryngeal cancer, non-small cell lung cancer, prostate cancer, nasopharyngeal carcinoma and ovarian cancer [32]. Moreover, a high expression level of hsa-miR-23a was associated with poorer overall survival in digestive system cancers [33]. Serum overexpression of hsa-miR-23a has been identified in cancer types like breast, gastric, esophageal and colon carcinoma. However, the expression, function, and exact role of hsa-miR-23a-3p in thyroid cancer have not yet been validated yet, neither in tumor tissue nor in serum.

In this study, we found that hsa-miR-23a-3p is upregulated in the serum of patients with PTC. There is only one study so far in which researchers found that hsa-miR-23a was significantly downregulated in PTC tissue samples compared with adjacent tissues. Especially, upregulated expression of hsa-miR-23a may significantly inhibit PTC cell proliferation, promote apoptosis, and induce cell cycle arrest in the G0/G1 phase [34].

We additionally observed the numerical downregulation of miR-17a-3p, a member of the miR-17-92 cluster, previously implicated in thyroid tumorigenesis [35]; the role of miR-17-3p in PTC remains poorly defined. MiR-17-3p has not been reported among the most significantly deregulated miRNAs in the literature. This lack of consistent evidence suggests that miR-17-3p may not represent a central driver of PTC biology, although its contribution as a context-dependent or secondary regulatory miRNA cannot be excluded and warrants further investigation.

We also observed discrepancies with a number of measured miRNAs previously described in the literature. In a recent review the expression of hsa-miR-130b-3p, hsa-miR-17, hsa-miR-150-5p and hsa-miR-200c-3p in thyroid cancer has been the subject of numerous studies elucidating the pathways involved in PTC [19]. hsa-miR-200c-3p appears to be a tumor suppressor, and our findings, suggestive of downregulation, may be consistent with this function. hsa-miR-296-5p was among the miRNAs upregulated in PTC; however, in our study hsa-miR-296-5p was downregulated in accordance with Zhou et al. [36]. Similarly, Park et al. revealed that over expression of hsa-miR-21 could be used to differentiate thyroid cancers from benign tumors, however the upregulation of hsa-miR-21 in our PTC cohort did not reach statistical significance [37]. The downregulation of hsa-miR-885-5p has been investigated by Jin et al. They suggested that upregulated hsa_circular RNA_0004458 contributes to progression of PTC by inhibition of hsa-miR-885-5p [38]. The expression of hsa-miR-130b-3p, hsa-miR-17, hsa-miR-150-5p, and hsa-miR-200c-3p in thyroid cancer has been the subject of numerous studies elucidating the pathways involved in PTC. hsa-miR-200c-3p appears to be a tumor suppressor, and our findings suggest its downregulation may be consistent with this function [39].

The present pilot study has several limitations that should be acknowledged. Heterogeneity in serum miRNAs in thyroid cancer, as in other cancers, is a multifaceted issue influenced by patient genetic and epigenetic factors, technical variability, and other clinical parameters. The small cohort size, combined with the large number of miRNAs screened, limits statistical power does not allow for multiple hypothesis testing correction while the absence of external validation datasets limits the generalizability of the findings. An additional important limitation is the nonuniform distribution of sample numbers, patient age and gender in the study cohorts, as a result of RNA quality control and limited sample availability. However, the predominance of female patients observed in our cohort reflects the well-established female predominance of thyroid cancer, with reported female-to-male ratios of approximately 3:1 [40]. Additionally, the age distribution of the PTC cohort was significantly higher than the control consistently with the epidemiological characteristics of thyroid cancer reported in the literature, where the peak incidence occurs between the fourth and sixth decades of life [40]. Therefore, the results should be interpreted as preliminary and hypothesis-generating. Future studies in larger and more balanced cohorts, including examination of preoperative versus postoperative samples and aggressive PTC variants such as diffuse sclerosing variant (DSV), tall cell variant (TCV), columnar cell variant (CCV), solid variant (SV), and hobnail variant (HV), will be necessary to validate and further explore these candidate miRNAs.

## 5. Conclusions

We conducted initial screening of 84 serum miRNAs in patients with PTC and benign thyroid nodules to identify potential noninvasive biomarkers. Despite these limitations, this exploratory pilot study identified candidate circulating miRNAs showing differential expression between PTC and benign thyroid disease. The next critical step is to validate the clinical relevance of hsa-miR-23a-3p and hsa-miR-574-3p in larger, independent cohorts of PTC patients.

## Figures and Tables

**Table 1:** Demographic data and clinicopathological characteristics of the patients passed quality control.

Variables	PTC Group (Group 1) (*n* = 8)	Benign Thyroid Disease (*n* = 6) Control Group
Age in years at diagnosis	46.6 (29–55)	29.8 (18–49)
Gender		
Female	7 (87.5%)	4 (66.67%)
Male	1(12.5%)	2 (33.33%)
Tumor size		
>1 cm	1 (12.5%)	
<1 cm	7 (87.5%)	
Number of malignant foci		
Single	2	
Multiple	6	
pT feature		
T1	4	
T1a	3	
T1b	1	
pN feature		
N0	6	
N1 <1 cm >1 cm	2 0	

**Table 2:** Fold changes in miRNA expression in PTC serum compared to benign thyroid disease serum.

Mature ID	*p*-Value	Fold Change *
**hsa-miR-574-3p**	**0.032699**	**0.3749**
hsa-miR-150-5p	0.273964	2.789
hsa-miR-23a-3p	0.055960	2.7214
hsa-miR-21-5p	0.149505	2.3657
hsa-miR-17-3p	0.072766	0.4878
hsa-miR-296-5p	0.209467	0.4854
hsa-miR-885-5p	0.433342	0.4442
hsa-miR-130b-3p	0.110563	0.3935
hsa-miR-200c-3p	0.199865	0.3545
hsa-miR-17-5p	0.146503	0.2964

* miRNAs with *p*-values < 0.05 are included in the table above the bold line. * miRNAs with *p*-values between 0.05 and 0.1 are highlighted in red in the table as falling within the marginal significance zone.

## Data Availability

The original contributions presented in the study are included in the article/Supplementary Materials. Further inquiries can be directed to the corresponding author.

## Supplementary Materials

The following supporting information can be downloaded at https://www.mdpi.com/article/10.3390/biology15080626/s1: Table S1: List of 84 miRNAs included in the PCR array panel; Table S2: Differential expression of 84 serum miRNAs between PTC and Control groups; Table S3: Analysis of miRNA expression in an age-matched cohort of Control and PTC groups.
