# Supplementary material for: Disease-Relevant Preoperative Serum miRNA Levels in Papillary Thyroid Cancer
Source: Biology (Basel). 2026 Apr 16;15(8):626. doi: 10.3390/biology15080626 (PMC13114036; doi:10.3390/biology15080626)
Supplement: Supplementary file 1 [file biology-15-00626-s001.zip › Supplementary.Table S3.pdf]

Supplementary Table S3. miRNAs showing >2fold change in expression in PTC sera compared to benign thyroid disease sera highlighted in Table 2 in age-matched cohort.

Mature ID	P-value	Fold Change
hsa-miR-574-3p	0,02633	0,334
hsa-miR-150-5p	0,36772	3,069
hsa-miR-23a-3p	0,056935	2,6009
hsa-miR-21-5p	0,090119	3,6119
hsa-miR-17-3p	0,022821	0,367
hsa-miR-296-5p	0,096635	0,3154
hsa-miR-885-5p	0,296185	0,3472
hsa-miR-130b-3p	0,053366	0,283
hsa-miR-200c-3p	0,15126	0,4889
hsa-miR-17-5p	0,004551	0,3661
